# Utilization of Obstetric Analgesia for Labor Pain Management and Associated Factors among Obstetric Care Providers in Public Hospitals of Addis Ababa, Ethiopia: A Cross-Sectional Study

**DOI:** 10.1155/2021/9973001

**Published:** 2021-11-22

**Authors:** Rediet Gido, Tesfaye Assebe Yadeta, Abera Kenay Tura

**Affiliations:** ^1^Department of Midwifery, College of Health Science and Medicine, Dilla University, Dilla, Ethiopia; ^2^School of Nursing and Midwifery, College of Health and Medical Sciences, Haramaya University, Harar, Ethiopia; ^3^Department of Obstetrics and Gynaecology, University Medical Centre Groningen, University of Groningen, Groningen, Netherlands

## Abstract

**Background:**

In low-income countries, pain-free labor initiative is an emerging concept and still parturient undergoes through painful labor; this is not different in Ethiopia; despite the national direction to use analgesia for labor pain and strong demand from the women, evidence on utilization of obstetric analgesia for labor pain management in Ethiopia is scarce. The objective of this study was to assess level of obstetric analgesia utilization and associated factors among obstetric care providers in public hospitals in Addis Ababa, Ethiopia.

**Methods:**

An institution-based cross-sectional study was used. All obstetric care providers working in labor and delivery units in public hospitals in Addis Ababa were included. The data were collected using a self-administered structured questionnaire. After checking for completeness, data were entered into Epi-data 3.1 and analyzed using SPSS 20. Bivariate and multivariable logistic regressions were used to identify factors associated with utilization of obstetric analgesia.

**Result:**

Of 391 obstetric care providers included in the study, 143 (36.6%; 95% CI: 31.5–40.9%) reported providing labor analgesia. Having adequate knowledge (AOR 2.7; 95% CI: 1.37–5.23), ten and more years of work experience (AOR 4.3; 95% CI: 1.81–10.13), and availability of analgesics (AOR 3.3; 95% CI: 1.99–5.53) were significantly associated with providing labor analgesia.

**Conclusion:**

Slightly more than 3 in 10 obstetric care providers reported providing labor analgesics to women. Training of providers and ensuring adequate supply of analgesics is required to make sure that women in labor would not suffer from labor pain.

## 1. Background

Although labor is a natural physiologic phenomenon, it is accompanied with severe pain which requires management [1]. Labor pain management with use of pharmacological agents or nonpharmacologic means has become an integral component of labor management [2]. Pain management in labor involves a series of activities that include pain assessment as well as pain control or abolition. Thus, obstetric care providers have a duty to utilize safe and optimal pharmacological interventions in attaining painless labor [3].

Women who are offered analgesics had greater birth satisfaction and healthy reproductive outcomes by addressing both the emotional and physical aspects [4]. However, inadequate labor pain management negatively impacts maternal and fetal wellbeing, as well as progress of labor [5]. It may further lead mothers to postpartum depression, posttraumatic stress disorder, negative experience and dissatisfaction after childbirth, and fear of childbirth, which increases maternal request for cesarean section [6–9].

In high-income countries, pain relief is part of routine labor management and more than 70% of women got obstetric analgesia [10, 11]. However, in several low-income countries, including Ethiopia, pain-free labor initiatives are new and level of its utilization remained largely unexplored [12]. The Ethiopian Food, Medicine, and Health Care Administration and Control Authority (FMHACA) recommended healthcare providers to give analgesics and anesthetics for labor management [13]. However, level of its utilization among obstetric providers is not yet well explored.

Although lack of analgesic drugs, healthcare delivery systems or knowledge, attitude, and skills of providers may affect utilization of labor analgesia [14, 15]. Given the national direction to use analgesia for labor pain and considering relief of labor pain as right of laboring women, assessment of provision of labor analgesia among obstetric providers is essential. This study was conducted to assess utilization of obstetric analgesia among obstetric care providers in major public hospitals in Addis Ababa, Ethiopia.

## 2. Materials and Methods

### 2.1. Study Setting

This is an institution-based cross-sectional study; the structured questioner-based study was conducted in labor and delivery units in public hospitals in Addis Ababa, the capital city of Ethiopia, located at 9° 10 48″ North and 38° 440 24″ East with a total land area of 54,000 hectare, divided into 10 subcities and 100 kebeles (lowest administrative units in Ethiopia). During the study period, there were 54 hospitals: 13 governmental, 39 private, and 2 nongovernment organizations hospitals. The study was conducted from February 25 to March 24 2020 in 10 public hospitals, where more than 7500 healthcare providers were working.

### 2.2. Study Population

Four hundred twenty-seven obstetric care providers working only in labor and delivery unit were invited, and a written informed consent was obtained after clarification of the purpose of the study, the guidelines to complete the questionnaire, ensuring confidentiality of the responses, and the right to withdraw the results at any time for no reason. Out of the 427 obstetric care providers approached, 391 of them returned completed questionnaires.

### 2.3. Sample Size and Sampling Procedure

The minimum sample size required for the study was estimated using the single population formula with the following assumptions: utilization of pharmacologic analgesics (45.2%) [16], 95% CI, and margin of error of 5%. The calculated sample size was 416. But, considering the overall small number of obstetric care providers working in labor and delivery units (*N* = 427), we included all eligible providers in the selected hospitals.

### 2.4. Data Collection Method

A structured, self-administrated questionnaire was conducted individually. Written consent was asked for voluntary participation. Contact details of the principal investigator was provided in case a participant had questions or concerns that arose during the study period.

Tools used in the study were adapted from related literatures [16, 17]. The questionnaire consisted of four parts: sociodemographic conditions, perception about labor pain, institutional-related questions, and use of labor analgesics. The questionnaire was pretested on 20 obstetric care providers working in Bishoftu General Hospital to measure the content validity of the tools in determining the clarity of questions, the effectiveness of the instructions, the completeness of response sets, the time required to complete the questionnaire, and the success of the data collection technique; results obtained were useful in appraisal and modification of the tools; these subjects were not from the study sample.

### 2.5. Data Processing and Analysis

Data consistency was checked and entered into Epi-data version 3.1, was exported to SPSS version 20 for further analysis, and was summarized by using descriptive statistics. Tables were used for data presentation. Bivariate logistic regression was used to identify factors associated with utilization of obstetric analgesia among obstetric care providers based on adjusted odds ratio (AOR), 95% CI, and *p* value <0.25.

We used the enter approach in for inclusion into the multivariate model, while the Hosmer–Lemeshow statistic was used for model diagnostics. The multivariable logistic regression model was used to control the possible effect of confounders, and finally, the variables that had independent association with utilization of obstetric analgesia were identified on the basis of AOR, with 95% CI and *p* value ≤0.05. The variables were entered to the multivariate model using the backward logistic regression.

### 2.6. Operational Definition

#### 2.6.1. Utilization of Labor Analgesia

Determined as a self-report of using labor analgesics to manage labor pain in the last delivery they attend [18].

#### 2.6.2. Knowledge

Knowledge-related question comprised of 9 items, and the score ranges from 0 to 9; knowledge was considered as adequate when obstetric care providers scores equal or above mean (i.e., ≥5.1) and considered as inadequate when they score below the mean [17].

#### 2.6.3. Attitude

Attitude-related question comprises 7 items, and possible response levels of all items were 5 (strongly disagree, disagree, uncertain, agree, and strongly agree), and total score ranges from 7 to 35. Then, attitude was considered as positive when obstetric care providers scores equal or above mean (i.e., ≥18.8) and considered as negative when they score below the mean after arranging all statements in affirmative way [17].

### 2.7. Ethical Consideration

This study was conducted in accordance with the Helsinki Declaration [19]. The study protocol was reviewed and approved by the Institutional Health Research Ethics Review Committee (Ref no.: IHRERC 017/2020) of College of Health and Medical Sciences, Haramaya University, Ethiopia. Permission to conduct the study in each hospital was obtained from Addis Ababa Health Bureau and respective hospital heads. Informed, voluntary, and signed consent was obtained from all study participants. Confidentiality of participants was maintained through using codes and anonymous questionnaire.

## 3. Results

### 3.1. Sociodemographic Characteristics

Of 427 obstetric care providers invited to the study, 391 (91.6%) returned the completed questionnaire and were included in the analysis. The mean age of the respondents was 28.7 (±3.8) years, ranging from 20 to 48. Majority of the respondents were 20–29 years old (66%), midwives (65%), and worked less than six years (61.4%). Almost half of the obstetric care providers are BSC holders (54.5%), work in specialized/referral hospitals (52.7%), and males (51.9%) (Table 1).

### 3.2. Utilization of Obstetric Analgesia

A total of 143 (36.6% (95% CI: 31.5–40.9%)) of obstetric care providers reported using labor analgesics to manage labor pain. Only 4.1% reported using routinely for labor management, while 21.5% and 11% reported using sometimes and up on maternal request, respectively. The most commonly used analgesics were pethidine 69 (48.3%), tramadol 60 (42%), and diclofenac 45 (31.5%) (Table 2).

### 3.3. Knowledge and Attitude toward Obstetric Analgesia

The most known pharmacologic method for managing labor pain were systemic opioids (181; 46.3%), nonopioid systemic analgesia (169; 43.2%), regional analgesia (129; 33%), inhalational (72; 18.4%), and cervical (53; 13.6%). Among the respondents, 36.1% asked laboring woman to provide pain relief. Similarly, 36.3% reported that pharmacologic labor pain management is the best method to manage labor pain. With regard to labor pain, 55.8%, 29.2%, and 15.1% described it as moderate, severe, and mild, respectively. Only 37.9% of the respondents reported to hear about the WHO pain ladder. In general, only 26.1% of the respondents had adequate knowledge about labor analgesics (Table 3).

Regarding attitude of obstetric care providers, 212 (54.2%) respondents believed that obstetric analgesia should be given for laboring mothers. In addition, 195 (49.9%) believe that labor pain of every mother should be managed. Two-thirds (67.5%) of the respondents stated that women should not endure labor pain. Although 60.4% believed that labor analgesia offers better birth experience and 58.6% had positive attitude toward obstetric analgesia, 36.6%, 37.1%, and 43.5% believe that it affects progress of labor, causes fetal distress, and late presentation, respectively (Table 4).

The main institutional-related reasons that impede utilization of analgesics in labor pain management routinely or at all were reported to be lack of training (231; 59.1%), absence of guidelines and protocols (226; 57.8%), lack of skilled obstetric care providers (221; 56.3%), unavailability of drugs (193; 49.4%), and cost of analgesics (83; 41.9%).

### 3.4. Factors Associated with Utilization of Obstetric Analgesia

Knowledge of labor analgesics, work experience, and availability of analgesics were found to be independently associated with utilization of obstetric analgesia. Providers with ≥10 year work experience were 4.3 (AOR: 4.3; 95% CI: 1.81–10.13) times more likely to use obstetric analgesia compared to those who practiced ≤5 years. Obstetric providers with adequate knowledge were 2.7 (AOR: 2.7; 95% CI: 1.37–5.23) times more likely to use obstetric analgesics compared to their counterparts. In addition, healthcare providers working in facilities where analgesics were readily accessible were 3.3 (AOR: 3.3; 95% CI: 1.99–5.53) times more likely to use labor analgesia compared to providers working in hospitals where analgesics were not readily available (Table 5).

## 4. Discussion

This study was conducted to assess utilization obstetric analgesia for labor pain management among obstetric care providers in public hospitals in Addis Ababa, Ethiopia. We found that almost 4 in 10 (36.6%) providers reported providing obstetric analgesics at least once in the last 12 months. Longer work experience, having adequate knowledge, and working in hospitals where analgesics were readily available were factors associated with utilization.

This finding is comparable with the study performed in Benin (38.9%) [20]. But, it is lower than findings from Egypt (70.1%) and Nigeria (49% and 48.9%) [15, 18, 21]. This variation might be due to differences in hospital protocols, availability of drugs and equipment, and availability of advanced health institutions and skilled health professionals. This could also be from sociocultural and socioeconomic variations. However, it is higher than the studies conducted in Bangladesh (7.4%) and East Gojjam zone, Ethiopia (8.4%) [22, 23]. The variation might be due to differences in perception of labor pain and its management [12]. This could also arise from variation in the study period, sample size, and differences in the study setting.

Although its side effects are a concern, congruent with other findings in Nigeria and Gahanna, pethidine was the commonest analgesics utilized [18, 24]. This may be because opioids are inexpensive and are simple to administer [25]. Regrettably, epidural analgesia was employed only by 12.6% of the participants. Although comparable with the findings from Egypt (12.4%) [21], this is lower than findings from USA (58%), Norway (25.9%), and UK (25%) [26–28]. This might be due to the limited number of skilled providers, unavailability of equipment, and lack of attention for labor pain management [17].

In line with findings from previous studies, providers with more years of experience were more likely to utilize labor analgesia [23, 29]. This may be due to the reason that as obstetric care providers became more experienced, their level of knowledge and attitude became improved [30]. In addition, having adequate knowledge was associated with utilization of labor analgesia. This is similar with studies conducted in Nigeria and Ethiopia [15, 23]. However, it is inconsistent with study performed in Kembata Tembaro Zone, Southern Ethiopia, which reported obstetric care providers with inadequate knowledge were practicing labor analgesia more likely [16]. This might be due to the differences in the supply of the analgesics which would be less in the peripheral settings compared to Addis Ababa where the pain-free labor initiative was initially launched and still underway. In settings where no supply exists, knowledge may not suffice for utilization. In addition, the variation in the study participants could result in variation. Our study included senior clinicians compared to the former study where majority of the participants were low-level cadres. This is supported by the study conducted in Australia showing that obstetricians prefer pharmacologic methods for labor pain relief [31]. Not surprisingly, availability of labor analgesics was significantly associated with utilization labor analgesics which is consistent with studies conducted in Ethiopia and Nigeria [15, 16].

## 5. Conclusion

Almost two in five obstetric care providers reported providing labor analgesia. Given the international practice and national direction toward pain-free labor, our findings are low. Providers with more years of work experience, having knowledge and working in hospitals where analgesics are readily available, were more likely to utilize labor analgesics. Ensuring adequate supply of analgesics and increasing awareness about importance of managing labor pain is essential to make sure that every woman would give birth without enduring labor pain, the most severe form of pain while giving life.

## Figures and Tables

**Table 1:** Sociodemographic characteristics of obstetric care providers working in labor and delivery in major public hospitals in Addis Ababa, Ethiopia, 2020 (*n* = 391).

Variables		*n*	%
Age	20–29	258	66
	30–39	117	29.9
	≥40	16	4.1

Sex	Male	203	51.9
	Female	188	48.1

Religion	Orthodox	224	57.3
	Muslim	90	23
	Protestant	71	18.2
	Catholic	6	15

Profession	Medical doctor	95	24.3
	Midwife	254	65
	Anesthetist	32	8.2
	Others^*∗*^	10	2.5

Qualification	Diploma	24	6.1
	BSc	213	54.5
	MSc	53	13.6
	General practitioner	69	17.6
	Obgyn (including residents)	32	8.2

Work experience	≤5	240	61.4
	6–9	93	23.8
	≥10	58	14.8

Level of hospital	General hospital	185	47.3
	Specialized/referral	206	52.7

Obgyn, obstetrician and gynecologist. Others^*∗*^, nurses and anesthesiologists.

**Table 2:** Types of labor analgesia offered by obstetric care providers in public hospitals of Addis Ababa, Ethiopia, 2020 (*n* = 143).

Types of labor analgesics offered		*n*	%
Systemic opioids	Pethidine	69	48.3
	Tramadol	60	42

Systemic nonopioids	Diclofenac	45	31.5
	Paracetamol	24	16.8
	Hyoscine	8	5.6

Regional analgesic	Epidural analgesic	18	12.6

**Table 3:** Knowledge on obstetric analgesia among obstetric care providers in public hospitals of Addis Ababa, Ethiopia, 2020 (*n* = 391).

Response		*n*	%
Know labor analgesics	Yes	242	61.9
	No	149	38.1

Analgesics methods	Systemic opioids	181	46.3
	Systemic nonopioids	169	43.2
	Regional analgesics	129	33
	Inhalational	72	18.4
	Cervical	53	13.6

Ever asked to provide labor pain analgesics	Yes	141	36.1
	No	250	63.9

Best method for managing labor pain	Nonpharmacologic	127	32.5
	Pharmacologic	142	36.3
	Slapping	15	3.8
	Nothing	120	30.7

Heard about WHO pain ladder	Yes	148	37.9
	No	243	62.1

Adequate knowledge	Yes	102 (26.1)	
	No	289 (73.90)	

**Table 4:** Attitude toward obstetric analgesia among obstetric care providers in public hospitals of Addis Ababa, Ethiopia, 2020 (*n* = 391).

Question	Disagree, *n* (%)	Neutral, *n* (%)	Agree, *n* (%)
Obstetric analgesia should be given for labor pain management	176 (45)	3 (0.8)	212 (54.2%)
Every laboring women should be managed with analgesics	174 (44.5)	22 (5.6)	195 (49.9)
Women should endure labor pain	264 (67.5)	44 (11.3)	83 (21.2)
Labor analgesia influences labor progress	207 (52.9)	41 (10.5)	143 (36.6)
Labor analgesia causes late presentation	171 (43.7)	50 (12.8)	170 (43.5)
Labor analgesia causes fetal distress	208 (53.2)	38 (9.7)	145 (37.1)
Labor analgesia offers a better birth experience	137 (35.1)	18 (4.6)	236 (60.4)
Overall attitude level	Positive		229 (58.6)
	Negative		162 (41.4)

**Table 5:** Factors associated with utilization of obstetric analgesia among obstetric care providers in public hospitals of Addis Ababa, Ethiopia, 2020.

Variables		Utilized		COR (95% CI)	AOR (95% CI)
		Yes	No		
Work experience	≤5	73	167	1	1
	6–9	32	61	1.2 (0.72–1.99)	1.25 (0.63–2.50)
	≥10	38	20	4.35 (2.37–7.98)	4.3 (1.81–10.13)^*∗*^

Profession	Midwives	67	187	0.28 (0.19–0.45)	0.51 (0.15–1.49)
	Others	76	61	1	1

Level of hospital	General	58	127	1	1
	Specialized/referral	85	121	1.54 (1.14–2.33)	1.41 (0.85–2.58)

Qualification	Lower level	7	17	1	1
	Medium level	55	158	0.85 (0.33–2.15)	2.3 (0.69–7.78)
	Higher level	81	73	2.7 (1.18–6.86)	2.7 (0.53–8.13)

Anticipation of labor pain	Mild pain	17	42	1	1
	Moderate pain	72	146	1.2 (0.65–2.29)	0.92 (0.44–1.955)
	Severe pain	54	60	2.2 (1.14–4.36)	1.7 (0.74–3.77)

Knowledge	Inadequate	77	212	1	1
	Adequate	66	36	5.05 (3.12–8.18)	2.7 (1.37–5.23)^*∗*^

Attitude	Negative	37	125	1	1
	Positive	106	123	2.9 (1.86–4.56)	1.5 (0.85–2.58)

Availability of analgesics	Not available	42	151	1	1
	Available	101	97	3.74 (2.41–5.81)	3.3 (1.99–5.53)^*∗∗*^

Received training	No	75	156	1	1
	Yes	68	92	1.54 (1.13–2.33)	1.13 (0.68–1.87)

^*∗*^ *P* value <0.05. ^*∗∗*^*P* value ≤0.001. Lower level, diploma; Mid-level, BSc; Higher level, MSc, general practitioners, consultants, and residents; Others, medical doctors, anesthetists, and anesthesiologists.

## Data Availability

The data used to support the findings of this study are available from the corresponding author upon request.
